# Position of Circulating Tumor Cells in the Clinical Routine in Prostate Cancer and Breast Cancer Patients

**DOI:** 10.3390/cancers12123782

**Published:** 2020-12-15

**Authors:** Gerit Theil, Paolo Fornara, Joanna Bialek

**Affiliations:** Medical Faculty, Martin Luther University Halle-Wittenberg, Clinic of Urology, 06120 Halle (Saale), Germany; paolo.fornara@uk-halle.de (P.F.); joanna.bialek@uk-halle.de (J.B.)

**Keywords:** prostate cancer, breast cancer, circulating tumor cells, treatment decisions

## Abstract

**Simple Summary:**

Many different therapies are applied to fight tumor disease. Blood-based biosources, like circulating tumor cells (CTCs), offer the opportunity to monitor the healing progression and the real-time response to the therapy. In this review, we analyze the outcomes of the clinical trials and scientific studies of prostate and breast cancer performed in the decade between April 2010 and April 2020. Additionally, we describe the abstracts from the 4th Advances in Circulating Tumor Cells (ACTC) meeting in 2019. We discuss the potential therapeutic opportunities related to the CTCs and the challenges ahead in the routine treatment of cancer.

**Abstract:**

Prostate cancer and breast cancer are the most common cancers worldwide. Anti-tumor therapies are long and exhaustive for the patients. The real-time monitoring of the healing progression could be a useful tool to evaluate therapeutic response. Blood-based biosources like circulating tumor cells (CTCs) may offer this opportunity. Application of CTCs for the clinical diagnostics could improve the sequenced screening, provide additional valuable information of tumor dynamics, and help personalized management for the patients. In the past decade, CTCs as liquid biopsy (LB) has received tremendous attention. Many different isolation and characterization platforms are developed but the clinical validation is still missing. In this review, we focus on the clinical trials of circulating tumor cells that have the potential to monitor and stratify patients and lead to implementation into clinical practice.

## 1. Introduction

Cancer is caused by multiple molecular alterations in normal host cells that act together to drive uncontrolled cell self-renewal, growth and invasion, and lead to malignant transformation and progression. The majority of cancer-associated deaths (approximately 90%) are induced by metastatic disease rather than the primary cancer [1]. The early detection of cancer and subsequent noninvasive tumor profiling and monitoring should be enabled in every cancer patient. Thus, there is an unmet clinical need for biomarkers to fulfill the claim in precision oncology.

Blood-based biosources such as circulating tumor cells (CTCs), cell-free DNA (cfDNA), tumor-educated platelets and cell-free nucleic acids (circulating tumor DNA, long non-coding RNA, messenger RNA and microRNA) offer this opportunity. These biomarkers, summarized as liquid biopsy (LB), could provide information on urgent cancer characteristics [2]. CTCs detach from primary or metastatic tumors to enter the bloodstream, from which a small CTC population has the ability to metastasize to multiple organs [3]. In addition, CTCs are genetically unstable, evading immune defenses and modification metabolism [3,4]. These characteristics reflect the dynamic and heterogeneous phenotype of CTCs. Furthermore, at present, we know that cancer cells survive after infiltrating distant organs and can be present for years in the bone marrow as disseminated tumor cells (DTCs), which are correlated with an increased risk of eventual clinical recurrence [5].

The concentration of CTCs in the blood is very low, and a single CTC is in the background of millions of blood cells. Nonetheless, CTCs could serve as a comprehensive window into metastatic disease for the real-time monitoring of therapy responses.

LB in the form of CTCs received tremendous attention following approval of the automated CellSearch^®^ system (Menarini Silicon Biosystems Inc, Huntington Valley, USA) by the Food and Drug Administration (FDA). Thus, the importance of CTC enumeration as a surrogate marker for survival benefits in breast and prostate cancer patients was commenced [6,7].

The clinical utility and reliable information of CTCs as useful biomarkers must still be demonstrated in the standard care of cancer therapy. In the latest guidelines (version 3) the Prostate Cancer Clinical Trials Working Group (PCWG) determined that for the outcome assessment of patients enrolled in clinical trials, the incorporation of CTC enumeration (using CellSearch platform) must be the endpoint [8]. This decision illustrates and emphasizes the importance of the serial biological profiling of cancer. Moreover, it promotes CTCs in the field of personalized cancer treatment, supplying unique information on individual cancer-associated variations in tumor burden.

In this review, we analyze current clinical studies and focus on the clinical application of CTCs in prostate and breast cancer patients. We outline important results of clinical trials that may be translated into clinical practice.

## 2. Evidence Acquisition

**Figure 1:**
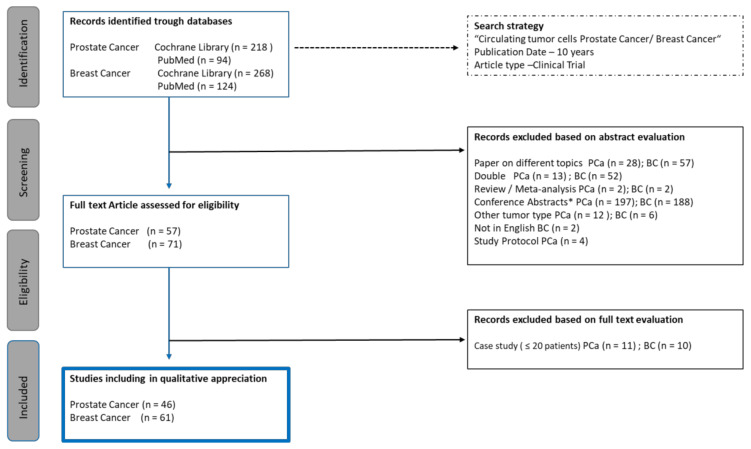
PRISMA flow diagram. BC = breast cancer; PCa = prostate Cancer; n = number; * other abstracts than ACTC (4th Advances in Circulating Tumor Cells) meeting 2019.

A literature review was performed via PubMed/Medline and the Cochrane Library (January 2010–April 2020). In addition, abstracts from the 4th Advances in Circulating Tumor Cells (ACTC) meeting in 2019 were searched for relevant abstracts. The search terms included CTCs, CTC, circulating tumor cell, circulating tumor cells, prostate cancer, breast cancer and clinical trial. All clinical trials reporting fewer than 20 patients were excluded (Figure 1).

## 3. Isolation Methods

The efficient capture of CTCs remains a technical challenge. Over the last decade, several platforms have been developed to detect these rare cells. CTC enrichment can be achieved based on physical and biological properties [9,10].

Currently, the most commonly used standardized method for detection and enrichment is the CellSearch^®^ system, based on affinity to epithelial cell adhesion molecule (EpCAM). The CellSearch^®^ system detects and enumerates cells expressing the epithelial marker EpCAM and cytokeratins (CK) 8/18+ and/or 19+, and the additional treatment of the blood samples with a CD45 antibody allows the elimination of leukocytes. A semiautomated fluorescence microscope captures images that are manually reviewed for the following CTC criteria: round to oval morphology, visible nucleus (DAPI positive), size > 4 µm, positive staining for cytokeratin, and negative staining for CD45 [11]. Recently, cells have been labeled with other markers, such as human epidermal growth factor receptor 2 (HER2), insulin-like growth factor 1 receptor (IGF-1R), epidermal growth factor receptor (EGFR), androgen receptor (AR), Ki-67 (a marker of proliferation) or vascular endothelial growth factor receptor 2 (VEGFR-2) [12].

Not all CTCs express EpCAM. It has been demonstrated that CTCs can undergo epithelial-mesenchymal transition (EMT) and can lose EpCAM expression. Therefore, analysis with the CellSearch^®^ system must be excluded, and other techniques based on physical properties must be employed. Mononuclear cells can be isolated by density gradient centrifugation using Ficoll or a more effective porous barrier. Cancer cells differ from normal cells in their electromagnetic charge. This feature is used during separation with dielectrophoretic field-flow fractionation (depFFF). Finally, most CTCs are larger than leukocytes and erythrocytes, and, as previously described, prepared buffy coat can be passed through porous membranes by selecting larger cells [13]. The Epic Sciences CTC platform uses only red blood cell lysates, and approximately 3 × 10^6^ nucleated cells are dispensed onto 10–12 glass micro slides and frozen at −80 °C until examination, allowing the unbiased detection and molecular characterization of CTCs [14]. The CTC-iChip also offers the opportunity for antigen-independent CTC isolation with negative depletion of normal blood cells. CTCs can be individually selected and analyzed as single cells suited for a detailed transcriptome analysis [15].

Numerous CTC isolation platforms were presented at the 4th ACTC meeting in 2019. It is not surprising that the CellSearch^®^ system was the most common platform used in the investigations. Interestingly, applications of the CellSearch^®^ system together with antibody-independent CTC isolation platforms in one blood sample were also common. An outstanding technology of a new in vivo approach, an indwelling intravascular aphaeretic CTC isolation system based on an EpCAM functionalized chip, was presented. Currently, the system has only been proven in a canine model [16]. Furthermore, many investigators utilized the physical properties of CTCs by label-independent isolation CTC methods (Isoflux—Fluxion Biosciences, Inc., Alameda, USA; Screencells—Sarcelles, France; Parsortix—ANGLE, Guildford, UK; ISET—Rarecells Diagnostics, Paris, France, and RosetteSep—STEMCELL Technologies, Vancouver, BC, Canada). The Vortex Biosciences VTX-1 instrument (Vortex Biosciences, Pleasanton, CA, USA) is a label-free CTC isolation system that enables the detection of gene expression in both CTCs and exosomal cfRNA from the same blood sample. An important issue is single-CTC isolation and analysis. For this purpose, different CTC isolation methods were combined. Workflows for single-cell selection using the QIAscout single-cell isolation platform were described. Pereira-Veiga et al. [17] used VyCAP (VyCAP BV, Enschede, Netherlands), DEPArry (Menarini Silicon Biosystems S.p.A, Bologna Italy) and manual micromanipulation for single-cell isolation. The authors concluded that the VyCAP Puncher system yielded a higher recovery rate and that the analysis at the single cell level provided better insights into CTCs’ heterogeneity [17].

## 4. Evidence Synthesis Prostate Cancer

It was estimated that over 1.3 million new cases of prostate cancer and 359,000 associated deaths worldwide occurred in 2018, accounting for the second most frequent cancer and the fifth leading cause of cancer-related death in men [18]. The disease phenotypes varied from indolent to aggressive. One challenge for clinicians is to determine the optimal sequencing therapies for patients who present intermediate, high-risk localized, locally advanced or metastatic prostate cancer (mPCa) to minimize overtreatment and improve outcomes. The treatment of mPCa is becoming increasingly complex [19].

In total, we screened 94 titles, and reviewed 46 full-text papers (Figure 1). From these 57 publications, 11 describe less than 20 and 46 describe 20 or more prostate cancer patients. Over 40 clinical trials evaluated the value of CTCs in metastatic castration-resistant prostate cancer (mCRPC) patients. There are only a few studies analyzing the value of CTC in new metastatic hormone-sensitive PCa (*n* = 1), neuroendocrine PCa (*n* = 1) and localized PCa (*n* = 3). In 10 phase III, 17 phase II and 10 phase I/II trials integrated measuring outcomes related to CTC enumeration or characterization.

The studies like SWOG S0421, MAINSAIL, COU-AA-301, analyzed the first-line docetaxel-base treatment with or without additional agents. The efficacy of therapy among the patients was determined enumeration of baseline CTC count and CTC count after defined cycle of chemotherapy or after treatment. The combination of CTC count with other serum markers were analyzed in the trials COU-AA-301, ELM-PC4 and IMMC38 as prognosis or surrogate biomarker for survival in mCRPC patients. Nine clinical trials evaluated androgen receptor (AR) splice variants (AR-Vs) in CTCs as marker responsible for castration-resistant prostate cancer progression. The baseline CTC-derived AR-V7 status as a biomarker of the response or resistance to therapies was analyzed in PROPHECY, TAXNEGY trial and three further monocentric trials.

In 18 studies, CTCs additional markers were analyzed: (1) standard genes (EpCAM, CK 8, 18, 19, and CD45-), (2) prostate-specific membrane antigen (PSMA)—protein, (3) osteoblast regulators—mRNA, (4) telomerase activity—mRNA, (5) TMPRESS2—mRNA, (6) AR-V7—mRNA/DNA. (Table S1).

### 4.1. CTC Enumeration—Prostate Cancer

Most clinical trials describing evaluations of the CTC count were published in the early 2000s. Multiple groups confirmed the prognostic value of the CTC count [20,21,22,23,24]. The SWOG S0421 trial validated the CTC count as a prognostic factor in mCRPC patients who received first-line docetaxel-based therapy. The CTC counts of 263 blood samples at baseline (day 0) and of 231 blood samples at day 21 were evaluated. It has been repeatedly acknowledged that a CTC count ≥5 per 7.5 mL at baseline determined with the CellSearch^®^ system was associated with a high tumor burden and poor disease outcomes. Additionally, a higher CTC count is correlated with worse bone pain, higher prostate-specific antigen (PSA) levels, more liver disease, lower hemoglobin levels and higher alkaline phosphatase levels [21].

A subgroup analysis from the MAINSAIL trial demonstrated that in mCRPC patients (*n* = 208), an increased CTC count from <5 cells/7.5 mL to ≥5 cells/7.5 mL after three cycles of docetaxel chemotherapy predicted significantly shorter overall survival (OS) (HR: 5.24, *p* = 0.025). A reduction in the CTC count from ≥5 cells/7.5 mL to <5 cells/7.5 mL was associated with the best prognosis (*p* = 0.003). Interestingly, there was no correlation of the baseline CTC count with the PSA level [25].

Heller et al. [26] determined the CTC count in combination with common prognostic laboratory measures (lactate dehydrogenase, LDH; PSA; hemoglobin, and alkaline phosphatase, ALK = ALPHA) in patients with CRPC. Their objective was to quantify a risk model to predict short-term versus long-term survival. For this purpose, data from patients enrolled in the phase III registration trial of abiraterone acetate (AA) plus prednisone (COU-AA-301; NCT00638690) and the registration trial of a similar design evaluating orteronel plus prednisone (ELM-PC4; NCT01193244) were used. The results suggested that the incorporation of CTC measurement into ALPHA as a prediction error of survival was 3.75 months (SE, 0.22) versus 3.95 months (SE, 0.28) for ALPHA alone [26]. In the COU-AA301 study (AA plus prednisone versus prednisone alone), the Prentice criteria [27] were also applied to test CTC counts and LDH as surrogates for OS at the individual mCRPC patient level. The combination of CTC count and LDH level satisfied the Prentice criteria and highlighted its clinical utility [28].

Furthermore, a baseline CTC count <5 cells was analyzed in mCRPC patients who were enrolled in the COU-AA301 and IMMC38 trials. Among 259 (50.7%) patients in the COU-AA301 trial and 212 (50.4%) in the IMMC38 trial, zero CTCs were detected at baseline. Patients were treated with an AR-targeting drug (COU-AA301) and chemotherapy (IMMC38) [29]. CTC progression was defined as any increase in the CTC count and conversion of a CTC count from <5 to ≥5 CTCs during the first 12 weeks of treatment. These data revealed that CTC progression is positively associated with poor OS. Interestingly, patients with CTC progression also had a significantly lower PSA response rate than those without progression [29].

The SWOG 0925 trial, demonstrated in metastatic hormone-sensitive prostate cancer patients treated with androgen deprivation therapy (ADT) ± cixutumumab (*n* = 105), that a low CTC count (0 versus 1 to 4 versus ≥5/7.5 mL blood) correlated with the PSA level [30].

A pooled analysis of five randomized trials (*n* = 4196 patients) investigated the CTC count as a predictor of prolonged survival in mCRPC patients. The CTC counts were determined by using the CellSearch^®^ system. The response measure endpoints were CTC0, CTC conversion and PSA levels at baseline and week 13. Patients who had a CTC count ≥1 at baseline and zero CTCs at week 13 were defined as CTC0. CTC conversion was defined as patients with a CTC count ≥5 at baseline and ≤4 at week 13. The results revealed that the use of the CTC0 count improved the ability to assess the treatment response [31].

Moreover, these results revealed that CTC conversion data from trials on treatment efficacy are highly reliable and can be obtained in a short time [26,31].

There are a marginal number of studies which investigate the role of CTC in localized prostate cancer patient in our search results. Murray et al. [32,33] examine the role of prostate cancer-specific CTC in patients after radical prostatectomy (RP) [32] and in patients with biochemical failure after RP [33]. The CTC were enriched by using density gradient isolation and detected by PSA immunocytochemistry. The author mentioned these CTCs as circulating prostate cells (CPCs) and postulated an identical phenotype to DTCs. They summarized that more CTC were detected in patients with positive margins, extracapsular extension, and vascular and lymphatic infiltration, which associated with biochemical failure [32]. The second publication of Murray and colleagues demonstrated a significant correlation between CPC detection and clinical variables such as progression-free survival (PFS) after a long follow-up period of 15 years. Additionally, CPC must express PSA and α-Methylacyl CoA racemase (P504S) and CD82 (tumor suppressor gene). Patients with CD82-positive CPC have a better biochemical failure-free survival at five years similar to CPC negative patients [33]. It is important to note from these results that blood samples (EDTA) were stored at 4 °C and analyzed within 48 h, which could be relevant for half-life of CPC, and also, no CPC number was provided.

### 4.2. Functional Characterization of CTCs—Prostate Cancer

The enumeration of CTCs has prognostic value in metastatic hormone-sensitive and metastatic castration-resistant prostate cancers. Nevertheless, CTCs still must demonstrate incremental value in predictive accuracy relative to known biomarkers. Therefore, the enumeration of CTCs has not yet become a standard in the clinic. Molecular phenotyping could aid in the investigation of prostate cancer-specific markers and be routinely incorporated into the care of patients with prostate cancer. CTC profiling can demonstrate the DNA, RNA or protein characteristics of pooled or single cells and reflect the real-time phenotype of the primary tumor or metastasis [34].

A potential predictive marker of sensitivity to the androgen biosynthesis inhibitor AA, the androgen-driven transmembrane protease serine 2 (TMPRSS2)–v-ets erythroblastosis virus E26 oncogene homolog (ERG), is the focus of several studies [35,36].

A proof of principle study described an analytically validated polymerase chain reaction (PCR)-based assay to detect TMPRSS2-ERG fusions in CTCs. However, the TMPRSS2-ERG fusion status in CTCs has a limited role as a predictive biomarker of sensitivity to AA in post chemotherapy-treated CRPC patients [37].

Additional effort has been directed at the molecular characterization of telomerase activity in CTCs. In men with mCRPC treated with first-line docetaxel ± atrasentan (SWOG 0421 trial), telomerase activity (TA) was investigated in live-captured CTCs in parallel to baseline CTC counts. The CTC TA measurement was performed with a slot microfilter. The authors analyzed TA in CTC lysates by qPCR-telomeric repeat amplification (TRAP) and concluded that CTC TA was an independent predictive marker for OS in men with a CTC count ≥5. Limitations of the TA assay were its low sensitivity; only 47% of patients with ≥5 CTCs captured by the CellSearch^®^ system showed prognostic TA [38].

The prostate-specific membrane antigen (PSMA) is overexpressed in prostate cancer including advanced stage [39]. Interestingly, the PSMA overexpression is correlated with higher tumor grade, androgen deprivation and increased in mCRPC patient [40]. A phase II clinical trial suggested the application of CTCs as a selection tool for the safety and efficacy of newly developed drugs. Patients with progressing mCRPC (chemotherapy-naïve; *n* = 42) were treated with docetaxel-encapsulating nanoparticles functionalized with PSMA molecules (BIND-014). The structure of this particle allows binding of PSMA-expressing tumor tissue or cells. CTC enumerations were performed with the CellSearch^®^ system and revealed that 39 of the 42 chemotherapy-naïve mCRPC patients were positive for CTCs. CTC conversion from an unfavorable count (≥5) to a favorable count (<5) was noted in 13 of 26 patients after treatment. The Epic Sciences platform allows the detection of nucleated cells after red blood lysis, and PSMA staining on CTCs and CTC clusters was subsequently used. In 16 (89%) of 18 patients with PSMA-positive CTCs detected, the number of CTCs was reduced after treatment. The PSMA expression levels can help select patients who are likely to benefit from PSMA-directed treatment. The authors concluded that PSMA expression on CTCs could serve as a patient selection biomarker for an early response in further clinical trials [41]. Another phase II trial investigated a PSMA antibody-drug conjugate (PSMA ADC) coupled to monomethyl auristatin E, which binds to PSMA-positive cells and induces cytotoxicity. The study demonstrated a decline in the CTC count of ≥50% in 78% (60/77) of mCRPC patients and conversion (from ≥5 cells/7.5 mL blood to less than 5 cells/7.5 mL blood) in 47% (36/77) of mCRPC patients. The highest CTC response (≥50%) was documented in patients with combined high PSMA expression on CTCs and low levels of neuroendocrine markers (94%) [42].

Armstrong and colleagues investigated treatment with radium-223 in men with bone metastases and progressive mCRPC in a small phase II trial. Evidence of prostate cancer osteomimicry biomarkers in CTCs was examined. These osteoblast regulators, such as bone alkaline phosphatase (B-ALP, gene ALPL), osteopontin, osteocalcin, osteoblast cadherin, runt-related transcription factor 2, bone gamma carboxyglutamate protein, tumor necrosis factor ligand superfamily 11, activator of NF-kappa-B ligand and secreted protein acidic and cysteine rich, and osteonectin, were examined at the RNA level. The authors concluded that genomic and phenotypic evidence supports osteomimicry in CTCs and tumor biopsies of men with mCRPC [43]. In multiple phase I/II studies, the CTC genotype or phenotype was used as a tool to determine possible direct drug targets or downstream drug targets [44,45,46,47].

AR splice variants (AR-Vs) represent a crucial mechanism responsible for castration-resistant prostate cancer progression. All variants have common structural features resulting from deletion of the ligand-binding domain (LBD) as a consequence of alternative splicing. Interestingly, the absence of the LBD can confer constitutive, androgen-independent activity [48]. AR-V7 is the most discussed splice variant in humans. A current study focusing on the mRNA analysis of CTCs revealed information on the clinical efficacy of anti-androgen treatment—CTCs which harbor the androgen receptor splice variant (AR-V7) and, despite the absence of the LBD, confer constitutive androgen-independent activity [48]. In 2014, Antonarakis et al. [49] analyzed the baseline CTC-derived AR-V7 status as a biomarker of the response or resistance to therapies in a small cohort of 62 men with enzalutamide- or abiraterone acetate (AA)-pretreated mCRPC. The results demonstrated that the outcomes of treatment with both drugs were significantly worse in patients who harbored the AR-V7 splice variant in CTCs than in those who did not harbor AR-V7. This study led to increased interest in the prospective utility of CTCs for the serial monitoring of second-generation AR antagonists as a mechanism of treatment resistance. Thus, CTCs might help to predict failure to treatment with enzalutamide and AA but not docetaxel or cabazitaxel [49,50,51].

The PROPHECY trial (ClinicalTrials.gov identifier: NCT02269982) compared two CTC platforms: the Johns Hopkins University modified-AdnaTest CTC AR-V7 mRNA assay and the Epic Sciences CTC nuclear-specific AR-V7 protein assay. The AdnaTest uses antibodies against EpCAM and HER2 for CTC capture. The Epic Sciences CTC platform uses only red blood cell lysates, and approximately 3 × 10^6^ nucleated cells are dispensed onto 10–16 glass micro slides. The prognostic significance of baseline CTC AR-V7 evaluated based on radiographic or clinical progression-free survival (PFS) in 118 men with high-risk mCRPC was determined. Both assays demonstrated significantly different PFS rates in AR-V7-positive men with mCRPC compared with AR-V7-negative men. Interestingly, the percentage agreement between the AR-V7 CTC assays was 82% (86/105). The authors concluded by two blood-based assays that the AR-V7 splice variant in CTCs may optimize treatment selection beyond the clinical assessment of prognosis [52].

In the phase II TAXYNERGY trial (ClinicalTrials.gov identifier: NCT01718353), Antonarakis et al. [53] analyzed an early taxane switch in men with chemotherapy-naïve, metastatic castration-resistant prostate cancer. The assumption was that the clinical response was associated with taxane drug-target engagement (DTE), which results in microtubule bundling (MTB) and nuclear AR localization (ARNL) in CTCs [53]. CTCs were isolated from PSMA-based microfluidic devices [54]. This study demonstrated that the combination of real-time CTC-based %ARNL and MTB mRNA detection at an early time point could be used to indicate a benefit in men treated with taxanes [53].

Furthermore, the nuclear localization of the AR-V7 protein in CTCs from 161 men with mCRPC was analyzed as a treatment-specific biomarker. The authors verified that nuclear localization of the AR-V7 protein was associated with superior survival with taxane therapy over androgen receptor signaling (ARS)-directed therapy (HR, 0.24; 95% CI, 0.10–0.57: *p* = 0.035) in a clinical setting [55]. The same group analyzed the phenotypic heterogeneity of CTCs (179 unique patients) to obtain supporting information for the treatment choice of androgen receptor signaling inhibitors (ARSIs) and taxanes in mCRPC patients. To quantify the heterogeneity of CTCs, the Shannon index was used. The phenotypic features were subdivided into 15 subtypes: “A”–“O”. For example, the following characteristics were associated with cell type A: low cytokeratin, no AR expression, and large cell size. The following characteristics were associated with cell type F: frequently in the histological cluster of 2 CTCs, AR expression and high cytokeratin expression. CTCs were processed with the Epic system. The authors concluded that low CTC phenotypic heterogeneity was associated with prolonged OS in patients treated with an ARSI. A higher Shannon index was associated with better OS for patients treated with taxanes [56]. The advantage of CTC analysis is to allow single-cell determination of nuclear AR-V7 protein localization in different CTC subtypes. In this context, Miyamato et al. demonstrated the RNA-based digital CTC quantification of prostate-derived transcripts as predictive of the AA response in men with mCRPC. Single-cell RNA analysis of CTCs was performed on the androgen-responsive transcripts PSA, KLK2, and TMRESS2, and on anterior gradient protein 2 homolog, PSMA, homeobox B13 and the androgen-independent transcripts FAT1 and STEAP2. The authors concluded that the digital scoring of CTC mRNA of prostate-derived transcripts can be used in high-throughput analyses in clinical practice [57].

In a second study, in a population of 34 localized prostate cancer patients prior to radical prostatectomy, Miyamoto et al. analyzed digital CTC quantification by whole transcriptome amplification and multiplex droplet digital PCR of a panel of 8 genes (AGR2, FAT1, FOLH1, HOXB13, KLK2, KLK3, STEAP2, TMPRESS2). Efficient risk stratification of localized PCa patients to guide optimal treatment by digital CTC quantification was only recently achieved by using the differential weighting of 6 genes from the panel, therefore, the authors could predict early presence or absence of prostate cancer dissemination in localized disease [57].

Among 108 posters, 16 reported the analysis of prostate cancer patients. Five abstracts from the ACTC meeting in 2019 discussed the possible roles of AR and splice variants in mCRPC patients. In this context, new quantification platforms for the proteins or mRNAs of CTCs were presented. Hofmann et al. [17] analyzed an mRNA in situ padlock probe to detect AR and AR-V7 on CellCollector. AR-V7 was detectable in 91% (10/11) of advanced prostate cancer patients. An analysis by Markou et al. [17] demonstrated an RT-qPCR assay for determination of the proto-oncogene PIM-1 mRNA in the EpCAM-positive fraction of mCRPC patients (*n* = 50). They concluded that PIM-1 mRNA expression should be further assessed to profile CTCs. CTC clusters (two or more CTCs) were evaluated in metastatic breast cancer (MBC) patients (*n* = 57) and mPCa patients (*n* = 57) in relation to PFS and OS. CTCs were captured with the CellSearch^®^ system. The authors confirmed the further prognostic value of the CTC cluster compared with the CTC count alone [17].

## 5. Evidence Synthesis—Breast Cancer

Breast cancer is one of the most common cancers among women worldwide, with an incidence of approximately 17,000,000 cases per year and the highest mortality among women [58,59]. In the early status, breast cancer is relatively treatable, whereas metastasis oft finishes with death of the patient. Even though decision on the proper therapy is not an easy step, one must consider the molecular subtype of the tumor.

Over 70 clinical trials analyzing CTCs in breast cancer patients have been performed. From these 71 publications, 10 describe less than 20 and 61 describe 20 and more patients bearing diverse breast carcinomas of various stages: 17 with primary breast cancer (PBC) and 24 with MBC. Forty-one studies were described as a part of clinical trials, or at least some of the patients were included in clinical studies (trial phase I, 2; II, 18; III, 15). The most frequently used system for the CTC isolation in breast cancer patients is the CellSearch^®^ system (*n* = 47 trails). Patients bearing primary breast cancer were analyzed in several studies like SUCCESS, NeoALLTO, and BEVERLY-2. The efficacy of the given therapy among the patients was examined by enumeration of CTCs before and after applied study-specific therapies (patients included in SUCCESS, BEVERLY-2) as well as by evaluating and comparing the rate of pathological complete response (pCR) in HER2/ErbB2-overexpressing and/or HER2/ErbB2-amplified PBC (patients included in NeoALLTO). In two studies, analysis of additional staining of CTCs (Barriere et al.) or additional factors in blood (patients included in SUCCESS I) were performed. Changes of CTC number and/or phenotype by metastatic breast cancer (MBC) patients were analyzed in other trials. Six of them (OnSITE, CirCe01 and some multi- or mono-centric studies) focused only on analysis of the enumeration, when the others (CAMELLIA, TBCRC, NEOZOTAC side-study, LANDSCAPE, BCA2001, AVALUZ) investigated phenotypical changes among CTCs after applied therapies.

In 27 studies, CTCs’ additional markers were analyzed: (1) tumor markers (MUC1 and HER2), (2) subsidiary markers of stemness (CD44 and BMI1), (3) EMT markers (TWIST, AKT2, PI3KA, ALDH1, and vimentin), (4) apoptotic genes expression, (5) study-related genes (AGTR1), and (6) 55-CTC-specific genes (Table S2).

### 5.1. CTC Enumeration—Breast Cancer

The basic analysis of CTCs is their enumeration. Most investigators have noted the prognostic significance of the CTC counts for OS [60,61] as well as chemotherapy [62,63,64,65,66,67,68,69]. In analysis performed by Trapp et al. [70], describing the early-stage high risk PBC patients of the SUCCESS A trial, an association between the presence of CTCs two years after chemotherapy with zoledronate and shortened OS and disease-free survival (DFS) was observed. Symonds et al. [71] revealed that a decrease in CTC numbers from baseline to the first assessment after employing nab-paclitaxel and bevacizumab therapy followed by maintenance therapy with bevacizumab and erlotinib by metastatic TNBC patients correlated with prolonged PFS and OS. Similar results were noticed in the OnSITE study analyzing the CTC number before and after the second cycle of treatment of the HER2-negative advanced BC patients pre-treated with anthracyclines and taxanes, who received eribulin as third-line chemotherapy show similar results [72]. Liang et al. [73] analyzed the number of CTCs as one of the efficacy parameters for the comparison of three therapy strategies for tumor cryoablation with natural killer (NK) cells therapy (I, cryoablation; II, cryoablation + NK cell therapy; III, NK cell therapy-Herceptin) and Herceptin for patients with HER2-overexpressing recurrent BC. The reduced number of CTCs after combined therapies correlated partially with prolonged PFS [73]. According to the prospective study with newly diagnosed metastatic BC patients treated with the systemic therapy, the detection of ≥5 CTCs or CTC clusters can predict a growing hazard ratio and worse PFS and OS during therapy [74]. Identification of one or more CTCs before PBC resection correlates with poor relapse-free survival and OS [75]. Similarly, Goodman et al. [76] observed longer OS, local recurrence-free survival (LRFS), and disease-free survival (DFS) in CTC-positive patients enrolled in the phase III SUCCESS study and from the National Cancer Database (NCDB), who underwent radiotherapy than in patients who did not. On the other hand, after comparing two therapies (anthracycline-containing chemotherapy and anthracycline-free chemotherapy), Schramm et al. [77] did not notice any differences in the number of CTCs between the therapies in the HER2-negative patients with early BC included in SUCCESS C study. Similarly, none or only the weak prognostic relevance of CTC count was demonstrated by Jueckstock et al. [78] and Hepp et al. [79] The authors employed node-positive or high risk node-negative BC patients of the SUCCESS A study before adjuvant taxane-based chemotherapy or patients of the SUCCESS study receiving the therapy based on fluorouracil, epirubicin and cyclophosphamide (FEC) followed either by docetaxel vs. by docetaxel supplemented with gemcitabine, respectively [78,79].

Among studies enrolling 50 (Lelievre et al. [80]) or 28 (Tokudome et al. [81]) patients, CTCs were detected in only 8 or 9 at baseline and in only 1 or 5 at the final analysis, respectively. Similarly, during an analysis of the influence of therapy on HER2-negative breast cancer patients, Gonzalez-Angulo et al. [82] found CTCs only in 9 (28.1%) patients at baseline and in 3 (13.6%) at the end of the 18-week study, whereas Agelaki et al. [83] found a decrease in the percentage of HER2-positive CTCs from 93.45% (total number of patients = 21) to 66.6% after the first cycle. Paoletti et al. [84] described a study with 45 estrogen receptor-positive (ER)/HER2-negative metastatic or locoregionally recurrent disease BC patients enrolled in the CTC analysis, but only 11 presented ≥5 CTCs. Similarly, Pierga et al. [85] analyzed 41 HER2-positive MBC patients displaying brain metastasis, but the number of patients with CTCs decreased from 20 (≥1 CTC) and 9 (≥5 CTC) at baseline to 11 and 3, respectively, at day 21. However, elevated levels of CTCs were noted at baseline in both groups as strong prognostic factors [84,85]. In contrast, an analysis of ER expression on CTCs in patients with positive primary tumors (*n* = 16) showed intrapatient heterogeneity, though the small number of included patients had the authors conclude the link to the mechanism of the escape from the endocrine therapy [86]. On the other hand, after an analysis of ER/HER2 expression on CTCs in small MBC and LABC/IBC patient cohorts (*n* = 36), Somlo et al. [87] suggested that the pilot trial may help to validate CTC-based targeted therapy.

### 5.2. Functional Characterization of CTCs—Breast Cancer

Molecular characterization of a tumor allows division into different subtypes: ER/PR-positive and HER2-positive. Each subtype responds to different therapies [88,89,90]. The other subtype does not express any of the receptors (triple-negative, TNBC) or respond to any of the hormonal target therapies [91]. However, many reports indicate that independent of tumor size, histopathological grade, ER/PR status or axillary lymph node involvement, the HER2 status of a patient can change [92]. First, a negative primary tumor may create positive metastases, which can influence the therapy decision [93]. Therefore, the classification of a tumor using biopsy is crucial. However, due to the location of the metastasis, biopsy is not always possible. LB is less invasive and can be used to monitor disease development. Even if HER2 expression among the CTCs within each patient is heterogeneous, revealing the strongly positive cells in blood samples allows us to suggest a positive HER2 tumor status. This was demonstrated with the study investigating the efficacy of neoadjuvant treatment on inflammatory BC (HER2-negative—BEVERLY 01 or HER2-positive—BEVERLY 02) with non-metastatic patients (M0) or prospective study with M1 BC [94]. The detection rate of CTCs and the determination of their HER2 status could be a good clinical strategy during treatment [93,95,96].

CTC investigations can also support the prediction of survival in combination with other factors. In their studies with inflammatory BC patients of the BEVERLY-2 survival data, Pierga et al. [97] revealed that the analysis of CTCs and pathologic complete response (pCR) is a good combination of parameters for creating a subgroup with a very good survival prognosis. Based on a comparison of two patient groups with early-stage breast cancer, CTC-positive and CTC-negative, included in phase I SUCCESS study (FEC therapy described previously), König et al. [98] demonstrated that the cytokine profile could also serve as a marker for CTC involvement in disease progression. Among the T-helper cell 2 cytokine (Th2) levels, interleukins 8 and 13 (IL-8, IL-13) were highly secreted by the CTC-negative group of patients negative for progesterone receptor. This correlation was not observed in CTC-positive patients. Similarly, there was an association between the IL-4 level and survival (patients who died had a high IL-4 level) in hormone receptor-negative patients in the CTC-negative but not CTC-positive group. Vilsmaier et al. [99] speculated that Th1 cytokines (Il-1α and Il-1β) are also involved in the release of CTCs in breast cancer patients. Conversely, in a similar patient cohort, (phase I SUCCESS), the expression of two vascular markers, soluble fms-like tyrosine kinase-1 (sFlt1) and placental growth factor (PlGF), in correlation to the CTC status, found their increased expression in CTC-negative patients was analyzed [99]. This suggests that the overexpression of both markers in tumor cells inhibits invasion of the decanted tumor cells into blood vessels [100].

Several trials analyzed apoptosis in CTCs. Negative breast cancer responds to apoptosis, triggering tigatuzumab treatment and Paoletti et al. [101] hypothesized the induction of apoptosis in CTCs among metastatic TNBC patients treated with tigatuzumab in the included studies. However, no prognostic effect was observed [101]. Other studies describing the influence of zoledronic acid (ZA, an inhibitor of tumor growth and inductor of apoptosis) infusion on the CTC value in patients with locally advanced BC (group 1) and those with bone metastasis only (group 2) revealed a decrease in the CTC number after 48 h; however, the difference was minimalized after 14 days. Additionally, an analysis of the apoptotic marker M30 in CTCs after 14 days showed increased levels of apoptotic CTCs [102]. Similarly, in their analysis with patients bearing progressive metastatic BC shared in the cohorts, depending on the treatment (endocrine therapy, texane-based or non-texane based chemotherapy). Smerage et al. [103] hypothesized that the presence of M30-positive CTCs was associated with a good prognosis, but the results of their studies after any of the therapies produced the opposite outcomes. In patients with high numbers of CTCs, high levels of M30-CTC correlated with a poor prognosis, while high CTC-Bcl-2 (B-cell lymphoma 2) levels were associated with a good prognosis [103].

Another mechanism that is strongly correlated with cell invasion is EMT. Analyses of this process are crucial since it leads to downregulation of the expression of epithelial markers such as EpCAM. Such a situation can result in false-negative findings considering EpCAM-based CTC isolation. Guan et al. [104] examined CTCs of the HER2-negative metastatic BC women participating in CAMELLIA trial. This phase III study analyzed the metronomic capecitabine chemotherapy vs. intermittent capecitabine maintenance therapy following the capecitabine complemented with docetaxel first-line chemotherapy. Examined CTCs were isolated with a method based on cell size, enumerated and expression of epithelial markers (EpCAM, CK8/18/19), and mesenchymal markers was analyzed (Twist and Vimentin). The investigation of Guan et al. [104] revealed that the group of patients who secreted EMT-CTCs experienced shorter PFS than the group of non-EMT-CTC patients. Additionally, HER2-negative patients demonstrated almost two times higher EMT-CTC counts than HER2-positive patients [104]. Barriere et al. [105] analyzed CTCs in patients with early breast cancer. They detected cells with dedifferentiated characteristics (mesenchymal phenotype, stem cell phenotype or both) in 37.6% of the analyzed patients, and the predominant epithelial phenotype was present in 8.75% of probes. They concluded that the molecular analysis of CTCs is more relevant than enumeration only [105]. An analysis of both epithelial and mesenchymal CTCs is also suggested as a good tool to predict the responsiveness to eribulin [106].

Among 108 posters, 25 described the analysis of breast cancer patients. Many presentations addressed the relation of CTCs with therapy. An analysis of blood samples from 36 BC patients showed a positive association between the level of EMT-CTCs before treatment and the effect of neoadjuvant therapy. Kallergi et al. [17] examined the expression of cytokeratin/C-X-C chemokine receptor type 4/transcription factor jun-B (CK/CXCR4/JUNB) in DTCs isolated from the bone marrow (BM) of 39 HR-positive, HER2-negative breast cancer patients and compared them to that in breast cancer cell lines. They concluded that DTCs expressing these proteins appear to create a subgroup of BC patients at high risk of relapse. After an analysis of epithelial-mesenchymal plasticity (EMP) of primary tumors and their CTCs, Hassan et al. [17] concluded that CTCs may provide important information regarding the progression of cancer. Strati et al. [17] performed an analysis on 100 patients with early breast cancer and 19 healthy donors, and confirmed the importance of CTCs as prognostic factors after detecting the overexpression of TWIST1 and stem cell transcripts. Considering the treatment selection in metastatic TNBC, Zang et al. [17] examined the association between the levels of CTCs and programmed cell death 1 ligand 1 (PD-L1) expression. They suggested this as a potential predictive therapy marker in metastatic triple-negative breast cancer. In addition, the copy number alteration (CNA) profiles of CTCs may be linked to OS. An analysis of the ERα gene (ESR1) (Tzanikou et al. [17]) and PIK3CA gene (Stergioupoulou et al. [17]) revealed that hot-spot mutations in single CTCs is also possible.

## 6. Perspectives of Real-Time Monitoring

Anti-tumor therapies are long and exhaustive for patients. Real-time monitoring of the healing process could be a useful tool to evaluate therapeutic responses. The application of LB for clinical diagnostics could improve sequence screening, provide additional valuable information on tumor dynamics (early response, mutation-based resistance to target therapy) and help personalize management for patients (Table 1).

This less invasive method allows the analysis of CTCs, among other factors. Furthermore, CTCs may offer the opportunity for the real-time monitoring of cancer progression. Additionally, CTCs are sources of DNA, RNA and protein, which provide information on tumor heterogeneity. A good opportunity for this approach is CTC single-cell analysis [34]. At the 4th ACTC meeting, some new approaches for single-CTC isolation were presented.

The first step of involvement of CTCs as prognostic and predictive biomarkers in the clinic was the approval of the CellSearch^®^ system for CTC enumeration. Within the scope of validation, it was calculated that a cutoff of ≥5 CTCs/7.5 mL blood of breast or prostate cancer patients is associated with an unfavorable prognosis [6,7]. The CellSearch^®^ system was the best validated CTC platform, as demonstrated by the large number of publications and the large number of analyzed patient samples. In our review, 8659 prostate cancer and 12,994 breast cancer patients were examined with the CellSearch^®^ system. In summary, most studies used the only FDA-approved technique to detect CTCs; however, this system also has limitations (EpCAM-based detection, sample size).

For a long time, the main applications of CTCs in clinical trials were based only on enumeration. Many investigators suggest that enumeration could be an independent prognostic factor of DFS or OS, or at least in combination with other agents [21,26,28,62,69,72,77]. The statement of the PCWG published by Scher et al. [8] confirmed the importance of CTC enumeration.

However, in breast cancer patients, many reports indicate no relation between the CTC number and therapy response [77,78,79]. In prostate cancer patients, the detection rate of CTCs is approximately 80%, but unfortunately, not all active progressive cancers have measurable CTCs in the blood [107].

The clinical trials demonstrated the possibility of real-time monitoring in metastatic cancer patients. Future studies must develop a gold standard in combination of CTC isolation/characterization technology for the ability to identify the best treatment. This is the main requirement for patients to benefit from early therapeutic intervention.

## 7. Discussion

Cancer cells can enter and are motile in blood circulation long before the tumor diagnosed. They offer a possibility for an early cancer diagnosis than standard diagnostics tools of imaging or biomarkers. The CTC analysis can provide insight into personalized cancer characteristics.

CTCs are relatively rare cells with a heterogenetic phenotype and are difficult to capture. At present, there are several platforms that can be used to capture or characterize CTCs. All of these platforms have advantages and disadvantages. The user must be able to decide which is the best and most suitable method for individual cancer patients. However, it is possible that in metastatic or late stage patients, no CTCs are detected. One reason for such conditions, could be the necrotic changes, or reduced tumor vascularization or extreme heterogenic CTC population. Another reason is the variation of the CTC frequency in a single blood sample, which also includes the short half-life of CTC and possible circadian rhythms of CTCs. Furthermore, it must be considered that phenotypic changes in CTC, in terms of epithelial-to-mesenchymal transition (EMT), reflects features of and results in downregulation of cell surface marker EpCAM.

The EMT process is also implicated in the generation of cancer stem cells (CSC), which are cells with abilities to self-renewal [108]. Such changes increase aggressiveness of the tumor cells, provoke their dissemination from the tumor and induce metastases. Detection of CSC as subpopulation CTCs and EMT-CTCs by patients would be crucial information for the treatment and potential therapy resistance.

A more precise analysis of CTCs would be applying the markers like Vimentin (EMT) or CD44 (CSC) at any level (DNA, RNA, protein) in regard to their clinical utility. The broader knowledge on intratumor heterogeneity and dynamic genetic and physiological changes in CTCs undergoing EMT or stem cell CTCs enforces continuous widening of the spectrum of specific markers to be analyzed.

The tumor-released cells can circulate cell-clusters composed of different CTC subpopulation. Only a few of the reviewed trials analyzed CTC-clusters additional to CTCs. The presence of CTC-clusters supported the previous diagnosis obtained with CTCs [41,74].

In our opinion, the crucial point of the efficient CTC analysis, enabling to include them into diagnostics, is their isolation. The combination of the EpCAM-based CellSearch^®^ system with antibody-independent CTC isolation platforms in one blood sample extends the best suitable CTC isolation method. This enables the isolation of all subpopulation of CTCs including CTC, EMT-CTC, CSC and can provide a precise outcome. Approbation of such a system or platform as an additional or supporting diagnostic tool would make the therapy more precise.

Such an analysis could be helpful to avoid the low response rate to therapy. A good example is the detection of the HER2 status in circulating breast cancer cells, which could be opposite to that in the primary tumor [93]. A combined analysis of additional factors, as described by Paoletti et al. [101] (multiparameter CTC-Endocrine Therapy Index (CTC-ETI)), supplies more information and may predict resistance to endocrine therapy. In preclinical studies, researchers analyzed the combined results of the enumeration and expression of ER, HER2 and Ki-67 in MBC patients and demonstrated strong analytical validity of the technique through intrapatient heterogeneity [84].

The molecular characterization of AR mRNA in CTCs and the detection of AR-V7 in CTCs can be used as a tool to guide treatment decisions for men with advanced prostate cancer [51,52]. The benefits for patients are that they are protected against the unnecessary side effects of ineffective treatments.

During their investigations, Smerage et al. [103] revealed that in patients with high numbers of CTCs after treatment, the number of cells did not change; thus, the number of apoptotic cells increased. High levels of M30-CTC (apoptotic cells) correlate with a poor prognosis, whereas high levels of CTC-Bcl-2 (anti-apoptotic cells) are associated with a good prognosis [103]. This is a very important observation, as the initiation of apoptosis in cells as a reaction to environmental stress leads to many morphological and biochemical changes, and probably to the production and secretion of substances that can be spread via the bloodstream to other organs, causing damage [109].

The signals from the tumor microenvironment (TME) and the microenvironment of CTC population can modify the protein pattern of disseminated cells, preparing it to create metastasis. One of the proteins induced by TME but also by chemotherapy is prostaglandin (PG)-endoperoxide synthase 2 (COX-2), which finally promotes the carcinogenesis and the rate of cancer recurrence, reducing the survival rate. COX-2 is implicated in the suppression of the apoptosis causing the resistance of tumor cells. The downstream signaling protein of the COX-2 is, among others, the Bcl-2, the anti-apoptotic marker increased in CTCs analyzed by Smerage et al. [107] (reviewed in [110]). Since both the positive and the negative signals may be initiated by the therapy, the monitoring of the treatment is crucial. LB, as the CTCs, is an excellent tool to analyze the changes during the long healing process. Although this hypothesis needs further investigation, if it is correct, it could bear significant consequences (Table 1). The critical point of preanalytical variables on LB in prostate cancer was also discussed in the plenary session of the ACTC 2019 meeting [17]; however, this issue is also applicable for other cancer types, such as breast cancer. Howerd Scher pointed out, in this session, the importance of introducing robust quality controls in all steps, such as pre-analytical procedures, and sample collection and processing, as well as analytical steps like molecular assay specification and bioinformatics algorithm, and data reporting. This standardization is absolutely necessary for its application into clinical routine. Alix-Panabieres [111] summarized in her research, the need for more intervention studies on the implementation of CTCs in the clinic. All abstracts at the 4th ACTC meeting described diverse proofs of concept in CTC isolation and characterization, and confirmed the heterogeneity in this field.

Cabel et al. [112] summarized an analysis of the utility of CTCs in clinical trials. They noted three main concepts of the studies: (1) CTCs serve as surrogate tumor material, (2) CTC enumeration can be used to monitor therapy, and (3) specific biological features of CTCs and their relation to metastatic spread. This conclusion is still relevant. The clinical validity of CTC enumeration by the CellSearch^®^ system is very high; however, its utility is still not standardized in the clinic and requires further investigations. Moreover, the clinical relevance of CTC characterization indicates the current therapeutic target HER2/ER in breast cancer patients and AR-V targets elucidates the resistance mechanism for prostate cancer patients. Even if there is a great need to identify predictive markers for therapy, it remains a great challenge to implement CTCs as a robust standard tool in the clinic.

## 8. Conclusions and Future Perspectives

LB, in the form of CTCs, is an excellent tool to monitor the disease development and the progress of the therapy. One must emphasize that different isolation methods may give disparate results; therefore, in the clinic, it is crucial to use systems of comparable validation. There is still a great need to develop and standardize the platform adequate to each tumor type. This crucial step can be achieved by a combination of already existing methods based on EpCAM detection and physical properties of the cells. A milestone would be an acceptance of LB in clinical diagnostic routine as a basic or at least as a supporting factor detecting disease progress. Application of additional staining markers on CTCs, specific for each type of the tumor, could help to monitor the changes of the tumor-derived cells during early-stage therapy and help to make a decision on continuing or stopping the therapy. However, as we know with the example of PSA, to become the useful tumor biomarker, the protein or factor must reach the high trust of the clinicians based on long positive experience. It was demonstrated in mCRPC that the CTC count is independent of the PSA level. In conclusion, the CTC count, in contrast to the PSA level, is not directly affected by ADT. Another clinical application of the cell count is the kinetic of CTC number, which reveal a higher discriminatory power for overall survival [26,31].

## Figures and Tables

**Table 1:** Specification feature of CTCs (circulating tumor cells) for potential clinical application.

Cancer Type	Characterization	Clinical Utility of CTCs Validated in Trials	Implementation in Clinical Practice	Reference
Prostate Cancer	AR/AR-Splice Variants	Prognosis Treatment Selection Therapy Monitoring Drug resistance	requires further evaluation	[49,50,51,52,53,55]
	PSMA	Therapy monitoring	basis for future evaluation	[41,42]
	Enumeration	Prognosis	potential clinical application	[20,21,22,23,24,25,26,27,28,29,30,31]
Breast Cancer	HER2	Therapy monitoring Prognosis	basis for future evaluation basis for future evaluation	[87,94,95,96,105] [105]
	EMT	Prognosis Therapy monitoring	basis for future evaluation requires future evaluation	[104] [105]
	Apoptosis	Therapy monitoring	basis for future evaluation	[101,102,103]
	Enumeration	Prognosis Therapy monitoring	potential clinical application requires future evaluation basis for future evaluation requires future evaluation	[60,61] [74,97,102] [87,93,101,102,103,104,105,106]

AR: androgen receptor, PSAM: prostate-specific membrane antigen, HER2: human epidermal growth factor receptor 2, EMT: epithelial-mesenchymal transition, CTC: circulating tumor cells.

## Supplementary Materials

The following are available online at https://www.mdpi.com/2072-6694/12/12/3782/s1, Table S1: Main clinical Trials for Prostate Cancer, Table S2: Main clinical Trials for Breast Cancer.
