# Supplementary material for: Position of Circulating Tumor Cells in the Clinical Routine in Prostate Cancer and Breast Cancer Patients
Source: Cancers (Basel). 2020 Dec 15;12(12):3782. doi: 10.3390/cancers12123782 (PMC7765455; doi:10.3390/cancers12123782)
Supplement: Supplementary file 1 [file cancers-12-03782-s001.pdf]

Supplementary Materials:

Position of Circulating Tumor Cells in the Clinical Routine in Prostate Cancer and Breast Cancer Patients

Gerit Theil, Paolo Fornara and Joanna Bialek

Table S1. Main clinical Trials for Prostate Cancer.

Prostate Cancer Typ	Clinical Trial	CTC functional characterisation additional to enumeration	Isolation technic other than CellSearch	Reference
mCRPC	SWOG S0421	Telomerase activity		[22], [38]
	MAINSAIL			[25]
	COU-AA-301			[26], [28], [29], [31]
	ELM-PC4			[26]
	IMMC38/AFFIRM/ELM-PC-5/ ELM-PC-4/ COMET			[30]
mCRPC	Phase II	PSMA-Expression	Epic Science	[41], [42]
	pharmacodynamic study radium-223	RNA expression of osteoblast regulators (ALPL, osteopontin, osteocalcin, OB-cadherin, RANKL, and SPARC)	RosetteSep	[43]
	Registered study	mRNA AR-VL and AR-V7	AdnaTest	[49], [50], [51]
	PROPHECY	mRNA AR-V7 vs nuclear specific AR-V7 protein assay	AdnaTest vs Epic Science	[52]
	TAXYNERGY	% AR nuclear localization + tubulin b	GEDI	[53]
	Registered study	nuclear expression of AR-V7	Epic Sciences	[55]
	Registered study	CTC mathematical phenotype identification and Shannon index	Epic Sciences	[56]
Hormone-sensitive PCA	SWOG 0925			[30]

Table S2. Main clinical Trials for Breast Cancer.

Tumor status	Clinical Trial	CTC functional characterisation additional to enumeration	Isolation technic other than CellSearch	Reference
PBC	SUCCESS			[70, 76, 77, 79, 99, 100]
		HER2		[92]
			density gradient,	[78]
	NeoALTTO	Her2		[96]
	BEVERLY-2	HER2 / no additional staining		[94] / [97]
	NEOZOL			[80]
	TREAT-CTC-Studie	HER2		[95]
	Registered study	MUC1, HER-2, TWIST1, AKT2, PI3KA, ALDH1, CD44, BMI1	AdnaTest	[105]
	Registered study			[75], [82]
MBC	OnSITE			[72]
	CAMELLIA	Vimentin and Twist	CanPatrol	[104]
	TBCRC	M-30		[101]
	CirCe01			[67]
	LANDSCAPE	CK19mRNA+		[85]
	Registered study	HER-2 /vimentin and pan-cytokeratin /HER-2 /HER-2, ER /whole genome amplification; ESR1 gene mutation analysis	immuno /Microfluidic Chip /AdnaTest vs. CellSearch /immuno-phenotype (FAST) /Immuno	[83] / [106] /[93] / [87]/ [86]
	Registered study	CK19mRNA+ / M30 and Bcl2 / M30/M65 / HorR/ HER2		[63]/ [103] / [102] /[61]
	Registered study			[65], [60], [68], [74], [64], [76]

Publisher's Note: MDPI stays neutral with regard to jurisdictional claims in published maps and institutional affiliations

© 2020 by the authors. Licensee MDPI, Basel, Switzerland. This article is an open access article distributed under the terms and conditions of the Creative Commons Attribution (CC BY) license (http://creativecommons.org/licenses/by/4.0/).
